# Prescription channeling of COX-2 inhibitors and traditional nonselective nonsteroidal anti-inflammatory drugs: a population-based case–control study

**DOI:** 10.1186/ar1488

**Published:** 2005-01-17

**Authors:** Yola Moride, Thierry Ducruet, Jean-François Boivin, Nicholas Moore, Sylvie Perreault, Sean Zhao

**Affiliations:** 1 Faculty of Pharmacy, Université de Montréal, Montreal, Canada; 2 Centre for Clinical Epidemiology and Community Studies, SMBD Jewish General Hospital, Montreal, Canada; 3 Joint Department of Epidemiology and Biostatistics, and Occupational Health, McGill University, Montreal, Canada; 4 Department of Pharmacology, Université Victor Segalen, Bordeaux, France; 5 Department of Pharmacoepidemiology, Pharmacia Corporation, Paepack, New Jersey, USA

**Keywords:** administrative health care databases, COX-2 inhibitors, nonsteroidal anti-inflammatory drugs, pharmacoepidemiology, prescription channeling

## Abstract

This pharmacoepidemiologic study was conducted to determine whether risk factors for upper gastrointestinal bleeding influenced the prescription of cyclo-oxygenase (COX)-2 inhibitors and traditional nonselective nonsteroidal anti-inflammatory drugs (NSAIDs) at the time when COX-2 inhibitors were first included in the formulary of reimbursed medications. A population-based case–control study was conducted in which the prevalence of risk factors and the medical histories of patients prescribed COX-2 inhibitors and traditional nonselective NSAIDs were compared. The study population consisted of a random sample of members of the Quebec drug plan (age 18 years or older) who received at least one dispensation of celecoxib (*n *= 42,422; cases), rofecoxib (*n *= 25,674; cases), or traditional nonselective NSAIDs (*n *= 12,418; controls) during the year 2000. All study data were obtained from the Quebec health care databases. Adjusting for income level, Chronic Disease Score, prior use of low-dose acetylsalicylic acid, acetaminophen, antidepressants, benzodiazepines, prescriber specialty, and time period, the following factors were significantly associated with the prescription of COX-2 inhibitors: age 75 years or older (odds ratio [OR] 4.22, 95% confidence interval [CI] 3.95–4.51), age 55–74 years (OR 3.23, 95% CI 3.06–3.40), female sex (OR 1.52, 95% CI 1.45–1.58), prior diagnosis of gastropathy (OR 1.21, 95% CI 1.08–1.36) and prior dispensation of gastroprotective agents (OR 1.57, 95% CI 1.47–1.67). Patients who received a traditional nonselective NSAID recently were more likely to switch to a coxib, especially first-time users (OR 2.17, 95% CI 1.93–2.43). Associations were significantly greater for celecoxib than rofecoxib for age, chronic NSAID use, and last NSAID use between 1 and 3 months before the index date. At the time of introduction of COX-2 inhibitors into the formulary, prescription channeling could confound risk comparisons across products.

## Introduction

Although randomized clinical trials have confirmed the advantage of cyclo-oxygenase (COX)-2 inhibitors over traditional nonselective nonsteroidal anti-inflammatory drugs (NSAIDs) with respect to gastrotoxicity [1-8], a large number of spontaneous reports have incriminated COX-2 inhibitors [9]. Numerous editorials and letters have been published that question the safety of these products [10-17]. The randomized clinical trial is the design best suited to determine drug efficacy, but it is inadequate for the evaluation of effectiveness, which applies to heterogeneous patient populations and patterns of drug use observed in a real life setting. In addition to pharmacological differences across products, the dosages used for the various indications [18] and past experience with the drug (through the 'depletion of susceptibles' effect) [19] account for differences in the risk of adverse effects. In an observational setting, such as postmarketing surveillance, the decision to prescribe one product over another is influenced by the characteristics of the patient, the prescriber and the health care system [20]. In the absence of randomization, it is consequently of utmost importance, when comparing the risks associated with individual drug classes, to determine whether the patient populations are indeed comparable.

The present study was conducted to compare the prevalence of selected risk factors for upper gastrointestinal bleeding among patients prescribed COX-2 inhibitors with those among patients prescribed traditional nonselective NSAIDs, and to compare the characteristics of patients prescribed celecoxib and rofecoxib, which are the two COX-2 inhibitors marketed in Canada at the time of the study.

## Methods

### Design

A case–control analysis was conducted in which the prevalence of selected gastrointestinal risk factors and medical histories of patients prescribed COX-2 inhibitors (the cases) were compared with those of users of traditional nonselective NSAIDs (the controls).

### Setting

The study involved prescriptions acquired through community pharmacies by members of the Quebec public drug program. Identification of eligible patients and acquisition of study variables were conducted via linkage with four administrative health care databases containing information on beneficiaries, health professionals, pharmaceutical services and medical services.

### Study population

The study targeted all ambulatory adult residents (aged 18 years or older) of the province of Quebec who were members of the public drug coverage program. In Quebec, coverage of prescribed medications was universal for all elderly residents (those aged 65 years or older) regardless of income as well as for all welfare recipients. The program was broadened in 1997 to include patients who do not have access to a private insurance program regardless of age. For everyone, the program now includes a deductible payment and a co-payment, with a monthly premium that depends on the beneficiary's income. In practice, the program includes the following segments of the population: the great majority of community-dwelling elderly persons (>94%), welfare recipients and patients younger than 65 years who do not have access to private insurance (e.g. the self-employed).

A sample of 100,000 drug plan members who received at least one celecoxib or rofecoxib prescription between 1 January and 31 December 2000 was randomly selected. A sample of 60,000 nonselective NSAID users was also randomly selected during the same time period, and patients who used low-dose aspirin (acetylsalicylic acid [ASA] ≤325 mg/day) only were excluded from the comparison group. The study population included both new (incident) users and longer time (prevalent) users. The status of patients with respect to being a user of COX-2 inhibitor or nonselective NSAID was determined at the end of the study year. Patients who had received both a COX-2 inhibitor and a nonselective NSAID were considered to be COX-2 inhibitor users. The index date was defined as the date of first dispensation of a COX-2 inhibitor or, for the traditional nonselective NSAID group, the date of the first dispensation of a nonselective NSAID.

The following inclusion criteria were applied: participants were required to have been a resident of Quebec for at least 2 years before the index date; and they were required to have had continuous coverage of medical and pharmaceutical services for at least 2 years before the index date. These criteria were verified through the beneficiary database.

### Study variables

The dependent variable was the prescription of COX-2 inhibitors (celecoxib or rofecoxib) or traditional nonselective NSAIDs. The independent variables were selected risk factors for upper gastrointestinal bleeding: patient demographic characteristics (age, sex); prescribed dosage; concomitant use of corticosteroids or anticoagulants; history of gastropathy (using four indicators: prior diagnosis of gastropathy, history of upper gastrointestinal procedures, prior dispensation of gastroprotective agents, and prior referral to a gastroenterologist); and prior history of NSAID use. Comparisons were controlled for prescriber specialty, patient overall health status (using the Chronic Disease Score [CDS]) [21], income level, past use use of low-dose ASA, acetaminophen, antidepressants and benzodiazepines, and time period.

### Risk factors for gastrointestinal events

Patient demographic characteristics included age, sex and income level, which were sought from the beneficiary database. For reasons of confidentiality, only age on 1 July 2000 was available. Income level was indirectly derived from the type of coverage (amount of deductible payment and co-payment), which was assigned to the patient based on their income.

History of gastropathy was assessed during the year before the index date through the presence of a diagnosis consistent with upper gastrointestinal bleeding in the medical services database. When present, this diagnosis was found to be reliable [22]. However, because it is not mandatory for the physician to be reimbursed, it is often missing. Consequently, three other markers were used: presence of an upper gastrointestinal procedure (e.g. gastroscopy, radiological examination) in the medical database; prior referral to a gastroenterologist, using physician specialty in the medical database; and prior dispensation of gastroprotective agents in the prescription database. Prescribed daily dosage of the COX-2 inhibitors and the traditional nonselective NSAIDs was derived from the dose per unit, quantity dispensed and prescribed duration. Daily dosages were subsequently categorized into low, standard and high, (for each product the dosage thresholds are listed in Table 1). Standard dosages were the recommended anti-inflammatory dosages. The threshold for low-dose corresponded to the maximum approved over-the-counter dosage, or, for products available on perscription only, dosages below the recommended prescribed anti-inflammatory dosage. High dosages were those above the maximum recommended anti-inflammatory dosage.

Details regarding the dispensation of acetaminophen, low-dose ASA, corticosteroids (excluding asthma-related drugs) and anticoagulants during the year before the index date were obtained from the prescription database. Past use of NSAIDs was assessed through records of the dispensation of these agents during the year before the index date. Patterns of use were defined using three categories of recency (last dispensation ≤1 month, >1 to 3 months, and >3 to 12 months before the index date). For recent users, two categories of duration of use were obtained: chronic (defined as at least one dispensation in each quarter of the previous year) and nonchronic (defined as less than one dispensation in each quarter).

### Covariables

Other variables may influence the prescription of NSAIDs and could act as confounders if they are also associated with risk factors for gastrointestinal events. Patient overall health status was assessed through records on medications dispensed during the year before the index date using the CDS [21]. Scores are weighted according to the number of different chronic diseases under treatment and the severity of the diseases. The CDS has been found to predict subsequent mortality and hospitalization rates. Because health status at the index date was the variable most likely to influence the physician's prescription, dispensing data for the year before were used for the calculation. Based on the distribution of scores, four categories were defined: 0, 1–4, 5–9 and ≥10. In addition, prescriptions of antidepressants and benzodiazepines were also considered to confirm the findings of a previous unpublished study that demonstrated an association between antidepressant and benzodiazepine use and prescription of COX-2 inhibitors. Prescriber specialty at the index date was determined from the prescription database.

Index dates were categorized into three time periods during the study year in order to account for differences in the date of entry of COX-2 inhibitors into the formulary of reimbursed medications (July 1999 and April 2000 for celecoxib and rofecoxib, respectively). The time periods considered were January–June, July–September and October–December 2000.

### Statistical analysis

The strength of the association between each patient characteristic and prescribed drug class was measured using odds ratios. The concomitant effect of patient characteristics was examined using multivariate logistic regression. Three models were used: COX-2 inhibitors as a class versus traditional nonselective NSAIDs, celecoxib versus traditional nonselective NSAIDs, and rofecoxib versus traditional nonselective NSAIDs. All data were analyzed using the SAS statistical package (SAS versions 6.12 and 8.0 for Windows; SAS Institute Inc., Cary, NC, USA). The level of statistical significance was set at 0.05 and the statistical uncertainty of the estimates was assessed using 95% confidence intervals.

### Ethical considerations

No patient or physician identifiers were provided to the researchers; only scrambled identifiers were used throughout the study. The study was approved by the Université de Montréal Health Sciences Ethics Committee.

## Results

After applying the selection criteria, 42,422 celecoxib, 25,674 rofecoxib and 12,418 traditional non-selective NSAID users were identified for the study. The characteristics of the study population are presented in Table 2. Because of the very large sample size, all differences were statistically significant and therefore *P *values are not reported. Patients treated with celecoxib were on average slightly older than those treated with rofecoxib or traditional nonselective NSAIDs, and a larger proportion of women were treated with COX-2 inhibitors as opposed to traditional nonselective NSAIDs. For each of the four indicators of prior history of gastropathy, there was a larger proportion of COX-2 inhibitor users with a positive history as compared to nonselective NSAID users. For all indicators used, the proportion was also greater for celecoxib than for rofecoxib. Very few patients had used anticoagulants during the year before the index date, but again the prevalence of use was greater for COX-2 inhibitors than for traditional nonselective NSAIDs.

Using the data presented in Table 2, we were able to determine that, overall, very few patients had used a nonselective NSAID for the first time during the month before the index date. The proportion of patients who had received their last NSAID prescription in the distant past (between 3 and 12 months before index date) was greater for celecoxib than for rofecoxib. Of the patients treated with rofecoxib, 72.8% had not received any NSAIDs during the prior year, which means that it was often used as a first treatment obtained under prescription. This proportion was lower for celecoxib (63.7%) and traditional nonselective NSAIDs (55.2%). Only 6.3% of rofecoxib users had received their last NSAID prescription between 1 and 3 months before the index date, as compared with 7.8% among celecoxib users and 15.7% among nonselective NSAID users.

The great majority of NSAIDs were prescribed by general practitioners (85.9% of traditional nonselective NSAIDs, 85.3% of celebrex and 88.3% of rofecoxib prescriptions). Dosage levels were highly heterogeneous across products. A large proportion of traditional nonselective NSAIDs were prescribed at dosages lower than those recommended for anti-inflammatory indications (22.1%) in comparison with celecoxib (3.4%) and rofecoxib (18.2%). Conversely, the majority of COX-2 inhibitors were prescribed at standard anti-inflammatory dosages (65.3% of celecoxib and 73.0% of rofecoxib prescriptions). A relatively high proportion of COX-2 inhibitors, especially celecoxib, were prescribed at dosages in excess of standard recommendations (31.2% of celecoxib and 8.8% of rofecoxib prescriptions). There was a strong correlation between dosage and age. For all products, the proportion of low dosages increased with age, and conversely the proportion of high dosages decreased with age (data not shown). This relationship was also found for overall health status; the higher the CDS, the higher was the proportion of prescriptions for low dosages (30.4% of all prescriptions were of low dosages for patients with a CDS 10+ versus 14.5% for those with a CDS of 0).

Results of the multivariate logistic regression are presented in Table 3. Increasing age and female sex were both associated with greater likelihood of receiving a COX-2 inhibitor. Compared with patients aged 18–54 years, older patients were more likely to receive a COX-2 inhibitor, but this association was greatly confounded by dosage category. Income level marginally influenced the choice of product; patients with lower income favoured the less costly traditional nonselective NSAIDs. According to crude odds ratio estimates, there was a positive association between each indicator of history of gastropathy and the probability of receiving a COX-2 inhibitor. However, when all the indicators were fitted simultaneously in the multivariate model, a history of gastrointestinal procedures was no longer significant; this finding is probably attributable to correlation between the various indicators. The analyses revealed an association between the CDS scores and the probability of receiving a COX-2 inhibitor, although no trend was observed.

Use of acetaminophen, corticosteroids, anticoagulants, antidepressants and benzodiazepines during the year before the index date were all associated with the prescription of COX-2 inhibitors. On the other hand, patients who had received low-dose ASA during the previous year were more likely to receive a traditional nonselective NSAIDs than a COX-2 inhibitor. Specialists were less likely to prescribe a COX-2 inhibitor than were general practitioners.

Results from the multivariate logistic regression models specific for celecoxib and rofecoxib are presented in Table 4. As shown, the strength of the association with gastrointestinal risk factors was significantly greater for celecoxib than for rofecoxib for age, past use of NSAIDs between 1 and 3 months before the index date, and recent chronic NSAID use. Point estimates of odds ratio for sex, other patterns of NSAID use, prior dispensation of gastroprotective agents, prior referral to a gastroenterologist, prior gastrointestinal procedures, prior use of antidepressants and benzodiazepines, and anticoagulants were greater for celecoxib than for rofecoxib, but the difference was not significant.

Because rofecoxib was only included in the list of reimbursed medications in April 2000, it was not available for half of the first time period, which explains its lower likelihood of being prescribed than nonselective NSAIDs (odds ratio 0.24, 95% confidence interval 0.22–0.26). However, for the second period (July–Sept) there was no significant difference between rofecoxib and celecoxib.

## Discussion

This study provides empirical evidence that channeling exists in the prescription of COX-2 inhibitors. Patients with risk factors for gastropathy were more likely to receive a COX-2 inhibitor than a traditional nonselective NSAID. Age, sex and history of gastropathy are well known independent risk factors for gastrointestinal bleeding, and it is therefore not surprising that they influenced prescribing practices. The effect of sex may be explained by greater use of over-the-counter NSAIDs in the past, not recorded in the databases, for the treatment of dysmenorrhoea. The effect of corticosteroids and anticoagulants is also not surprising, given that these drugs represent contraindications to the prescription of traditional nonselective NSAIDs. These findings are consistent with those obtained in a recent study conducted in a UK primary care setting [23] but they contradict those reported in a elderly Medicare population in the USA [24]. In the latter study it appeared that there was over-treatment with COX-2 inhibitors in patients without risk factors, and under-treatment in patients who had at least one risk factor. The effect of past NSAID use is more difficult to interpret because of the lack of data regarding reasons for discontinuation of NSAIDs. Although past NSAID use has been found to be associated with decreased incidence of gastrointestinal bleeding, the impact that such a 'depletion of susceptibles' effect may have on prescribing practices remains to be clarified. Regardless of the underlying mechanism, it can be concluded from these results that past NSAID use is likely to confound risk comparisons across drug classes because it is an independent risk factor for gastrointestinal problems as well as influencing prescribing practices.

Patients who had received acetaminophen in the past were more likely to switch to a COX-2 inhibitor than to a traditional nonselective NSAIDs. Patients who had received antidepressants and benzodiazepines were also more likely to receive a COX-2 inhibitor than a traditional nonselective NSAID. This empirical finding is difficult to interpret. It may be hypothesized that physicians may be more likely in general to prescribe newer agents to patients who are anxious. More studies are needed to explore further the interaction between patients and physicians in order to elucidate this issue.

Although there was an association between physician specialty and prescription of COX-2 inhibitors or traditional nonselective NSAIDs, the results did not confound the associations between patient characteristics and prescription practices.

Results for patient overall health status and prescribing practices were highly confounded by dosage. This suggests that, for the sickest patients, prescribing practices are largely determined by dosage rather than by drug class. Patients with a high level of comorbidity still receive traditional nonselective NSAIDs but at lower dosages. Such findings are likely to be time-sensitive because COX-2 inhibitors were just introduced into the Canadian market during the study period, and there might have been reluctance in the medical community to prescribe newer agents to sicker patients.

Comparisons between the two COX-2 inhibitors indicated that for several risk factors under investigation the channeling process is stronger for celecoxib than rofecoxib. However, these findings should be interpreted with caution because for several of the risk factors investigated the differences between products were not statistically significant. On the other hand, celecoxib was not always at a disadvantage; past chronic NSAID use, which, according to the depletion of susceptibles effect, places patients at a lower risk for upper gastrointestinal events [19], was associated with a greater probability of being prescribed celecoxib than rofecoxib.

Many risk factors for gastrointestinal bleeding could not be ascertained in this study, such as smoking status and alcohol use, which are known risk factors for gastrointestinal events and have also been found to influence prescribing practices [23]. Also, there were no data on indications but we controlled for dosage, which, according to Griffin and coworkers [18], is more likely to influence the risk of gastrointestinal bleeding than indication *per se*. Dosage had a very large impact on the results, and its exclusion would have produced spurious differences across products. There were no data on over-the-counter use of NSAIDs such as aspirin and ibuprofen. Therefore, it was not possible to explore the concomitant use of nonprescribed NSAIDs. Finally, data are generalizable only to recent use (in the previous year). We were not able to explore the impact of more distant history. Nevertheless, the use of retrospective data obtained from administrative databases allowed us to examine the various associations in a truly observational setting without influencing prescribing practices in any way. In addition, the large sample size allowed us to conduct comparisons across individual products.

## Conclusion

Our results provide empirical evidence that the introduction of a new class of medications into the market results in the channeling of patients at high risk for adverse effects. However, as shown by the present study, differences across individual products cannot be predicted from their order of entry into the formulary. Other factors, such as marketing strategies, play a major role as well. Neverthless, one may conclude that selective prescribing results in a positive association between risk factors and drug use, which could confound risk comparisons across products.

## Abbreviations

ASA = acetylsalicylic acid; CDS = chronic disease score; COX = cyclo-oxygenase; NSAIDs = nonsteroidal anti-inflammatory drugs.

## Competing interests

This study was funded through an unrestricted grant from Pharmacia Corporation. YM was a paid consultant for this study. JFB, TD, NM and SP declare that they have no competing interests. SZ was an employee of Pharmacia Corporation at the time the study was conducted.

## Authors' contributions

YM, as principal investigator of the study, designed and coordinated the study, interpreted study results, and wrote the manuscript. TD conducted the statistical analyses. JFB participated in the design of the strategy for the sampling of the study population and helped to draft the manuscript. NM assisted in the conduct of the statistical analyses and contributed to the interpretation of the study results. SP assisted in the review of the literature and determined the relevance of the study. SZ conceived the study and participated in its design. All authors read and approved the final manuscript.

## Figures and Tables

**Table 1:** Dosage categories for each product

Generic name	Low dosage (mg/day)	Standard dosage (mg/day)	High dosage (mg/day)
Celecoxib	≤100^a^	>100 to 200	>200
Rofecoxib	<25	25 to <50	≥50
Acetylsalicylic acid	≤1300	>1300 to <4000	≥4000
Diclofenac (including Voltaren + Cytotec = Arthrotec)	≤50	>50 to 100	>100
Diflunisal	≤500	>500 to 1000	>1000
Etodolac	≤300	>300 to 900	>900
Fenoprofen	<1800	1800 to 2400	>2400
Flurbiprofen	≤50	>50 to 200	>200
Ibuprofen	<1000	1000 to 1200	>1200
Indomethacin	≤50	>50 to 100	>100
Ketoprofen	≤50	>50 to 200	>200
Mefenamic Acid	<750	750 to 1000	>1000
Naproxen	≤550	>550 to 1100	>1100
Piroxicam	≤10	>10 to 20	>20
Salsalate	≤500	>500 to 1000	>1000
Tiaprofenic Acid	≤200	>200 to 600	>600
Tolmetin	≤600	>600 to 1200	>1200

^a^According to our references, 100 mg celecoxib would be considered a standard dose. However, because none of the patients were prescribed lower dosages, we included 100 mg as a low dose (in order to avoid a 0 cell).

**Table 2:** Characteristics of the study population

	Traditional nonselective NSAIDs (n = 12,418)	Celecoxib (*n *= 42,422)	Rofecoxib (*n *= 25,674)
Age (years)			
18–34	18.6	3.7	6.7
35–44	15.8	6.5	9.7
45–54	13.2	9.9	12.1
55–64	13.5	15.9	16.4
65–74	18.8	30.5	27.1
75–84	15.1	27.1	22.7
85+	4.1	6.5	5.1
Sex			
Female	55.4	67.4	65.5
Male	44.6	32.6	34.5
Income level			
Low	14.8	10.9	11.4
Nonlow	85.2	89.1	88.6
Dosage category			
High	11.6	31.2	8.8
Standard	66.3	65.3	73.0
Low	22.1	3.4	18.2
Prior diagnosis of gastropathy	3.6	7.7	5.0
Prior gastrointestinal procedures	2.0	4.5	2.7
Prior dispensation of gastroprotective agents	14.0	29.9	24.3
Prior referral to a gastroenterologist	2.8	6.0	3.9
History of NSAID use			
Recent, first time	3.0	2.2	2.8
Recent, chronic	6.6	4.3	4.2
>1 to 3 months	15.7	7.8	6.3
>3 to 12 months	19.4	22.0	14.0
No use	55.2	63.7	72.8
Anticoagulants	1.3	3.3	3.0
Corticosteroids	11.7	19.6	16.9
Benzodiazepines	23.3	38.2	33.0
Antidepressants	10.5	17.2	15.7
Chronic Disease Score			
≥10	5.2	10.3	8.2
5–9	19.6	28.6	25.4
1–4	27.9	33.6	32.6
0	47.3	27.6	33.8
Prescriber specialty			
General practitioner	85.9	85.3	88.3
Cardiology	1.0	0.4	0.3
Internal Medicine	2.3	3.3	2.1
Neurology	0.3	0.3	0.2
General surgery	1.4	0.8	1.0
Obstetrics/gynaecology	1.5	0.2	0.3
Orthopaedic surgery	1.2	3.4	3.2
Rheumatology	2.5	2.5	1.7
Other	2.8	3.8	2.9

Values are expressed as percentages. NSAID, nonsteroidal anti-inflammatory drug.

**Table 3:** Multivariate analysis of the factors associated with dispensation of selective COX-2 inhibitors versus traditional nonselective NSAIDs

	Crude OR (95% CI)	Adjusted OR (95% CI)
Age group (years)		
75+	3.17 (3.01–3.34)	4.22 (3.95–4.51)
55 to ≤74	2.86 (2.74–2.99)	3.23 (3.06–3.40)
18–54	Reference	Reference
Female sex	1.61 (1.52–1.66)	1.52 (1.45–1.58)
Income level (lower)	0.72 (0.68–0.76)	0.90 (0.85–0.96)
Prior diagnosis of gastropathy	1.93 (1.75–2.11)	1.21 (1.08–1.36)
Prior gastrointestinal procedures	1.94 (1.71–2.22)	1.09 (0.94–1.27)
Prior dispensation of gastroprotective agents	2.37 (2.25–2.50)	1.57 (1.47–1.67)
Prior referral to gastroenterologist	1.89 (1.69–2.12)	1.23 (1.08–1.39)
Prior history of NSAID use		
Recent, first time	2.39 (2.14–2.66)	2.17 (1.93–2.43)
Recent, chronic	1.58 (1.46–1.70)	1.21 (1.11–1.32)
>1 to 3 months	1.12 (1.06–1.19)	0.95 (0.89–1.01)
>3 to 12 months	0.93 (0.88–0.98)	0.84 (0.80–0.89)
No use in past year	Reference	Reference
Corticosteroids	1.72 (1.62–1.82)	1.16 (1.07–1.24)
Anticoagulants	2.53 (2.15–2.98)	1.56 (1.32–1.85)
Antidepressants	1.69 (1.59–1.80)	1.37 (1.28–1.46)
Benzodiazepines	1.87 (1.78–1.95)	1.20 (1.14–1.26)
Acetaminophen	1.85 (1.76–1.94)	1.41 (1.34–1.49)
Low dose ASA	0.85 (0.81–0.89)	0.56 (0.52–0.59)
Chronic Disease Score		
10+	2.88 (2.65–3.14)	1.26 (1.13–1.41)
5–9	2.21 (2.10–2.32)	1.28 (1.20–1.37)
1–4	1.88 (1.80–1.97)	1.26 (1.19–1.33)
0	Reference	Reference
Specialist (versus GP)	0.99 (0.94–1.04)	0.89 (0.84–0.94)
Dosage		
High dose	1.91 (1.80–2.03)	2.19 (2.06–2.33)
Low dose	0.40 (0.38–0.42)	0.29 (0.27–0.30)
Standard dose	Reference	Reference
Time period		
January–June	0.72 (0.68–0.76)	0.54 (0.51–0.57)
July–Sept	1.02 (0.95–1.09)	0.99 (0.92–1.06)
October–December	Reference	Reference

All covariables included simultaneously in the models are listed in this table; models were not adjusted for any other factors. ASA, acetylsalicylic acid; CI, confidence interval; COX, cyclo-oxygenase; GP, general practitioner; NSAID, nonsteroidal anti-inflammatory drug; OR, odds ratio.

**Table 4:** Multivariate analysis of the factors associated with dispensation of celecoxib and rofecoxib versus traditional nonselective NSAIDs

	Celecoxib	Rofecoxib
Age group		
75+	5.34 (4.96–5.75)	3.06 (2.83–3.30)
55 to ≤74	3.65 (3.45–3.87)	2.62 (2.46–2.78)
18–54	Reference	Reference
Female sex	1.55 (1.47–1.62)	1.45 (1.38–1.52)
Income level (lower)	0.95 (0.88–1.02)	0.83 (0.77–0.89)
Prior diagnosis of gastropathy	1.21 (1.07–1.37)	1.11 (0.97–1.27)
Prior gastrointestinal procedures	1.21 (1.02–1.42)	0.97 (0.81–1.16)
Prior dispensation of gastroprotective agents	1.59 (1.48–1.71)	1.51 (1.41–1.63)
Prior referral to gastroenterologist	1.27 (1.11–1.45)	1.19 (1.03–1.37)
Prior history of NSAID use:		
Recent, first time	2.25 (1.99–2.54)	2.02 (1.79–2.29)
Recent, chronic	1.68 (1.52–1.85)	0.75 (0.68–0.84)
>1 to 3 months	1.36 (1.26–1.45)	0.48 (0.44–0.52)
>3 to 12 months	0.85 (0.80–0.91)	0.81 (0.76–0.87)
No use in past year	Reference	Reference
Corticosteroids	1.11 (1.02–1.20)	1.20 (1.11–1.31)
Anticoagulants	1.61 (1.34–1.94)	1.48 (1.23–1.78)
Antidepressants	1.38 (1.28–1.48)	1.37 (1.27–1.48)
Benzodiazepines	1.18 (1.12–1.25)	1.15 (1.09–1.22)
Acetaminophen	1.39 (1.31–1.48)	1.37 (1.28–1.45)
Low-dose ASA	0.67 (0.62–0.71)	0.58 (0.54–0.62)
Chronic Disease Score		
10+	1.26 (1.11–1.43)	1.20 (1.06–1.37)
5–9	1.28 (1.19–1.38)	1.23 (1.14–1.33)
1–4	1.25 (1.17–1.33)	1.26 (1.18–1.34)
0	Reference	Reference
Physician specialty	0.96 (0.90–1.03)	0.83 (0.77–0.89)
Dosage		
High dose	3.36 (3.15–3.58)	0.76 (0.70–0.82)
Low dose	0.09 (0.09–0.10)	0.73 (0.69–0.78)
Standard dose	Reference	Reference
Time period		
January–June	1.25 (1.16–1.34)	0.24 (0.22–0.26)
July–September	1.09 (1.00–1.19)	0.93 (0.87–1.01)
October–December	Reference	Reference

ASA, acetylsalicylic acid; NSAID, nonsteroidal anti-inflammatory drug.
